# Defluorination and adsorption of tetrafluoroethylene (TFE) on TiO_2_(110) and Cr_2_O_3_(0001)

**DOI:** 10.1038/s41598-021-00952-w

**Published:** 2021-11-03

**Authors:** Jessiel Siaron Gueriba, Nur Ellina Annisa Salehuddin, Wilson Agerico Diño, Kiminori Washika, Hiroshi Nakamura, Tatsumi Kawafuchi

**Affiliations:** 1 Department of Applied Physics, Osaka University, Suita, Osaka 565-0871 Japan; 2 Department of Physics, De La Salle University, 2401 Taft Avenue, 0922 Manila, Philippines; 3 Institute of Laser Engineering, Osaka University, Suita, Osaka 565-0871 Japan; 4 Department of Chemical Sciences, Faculty of Science and Technology, Universiti Kebangsaan Malaysia, 43600 Bangi, Selangor Malaysia; 5 Center for Atomic and Molecular Technologies, Osaka University, Suita, Osaka 565-0871 Japan; 6 Laser Technology Laboratory, Hirotec Co., Ltd, 5-2-1 Ishiuchiminami, Saeki-ku, Hiroshima, 731-5197 Japan; 7 Research and Development Division, Technology Development Department, Charmant Inc., 6-8 Kawasari, Sabae, Fukui 916-0088 Japan

**Keywords:** Materials science, Condensed-matter physics, Surfaces, interfaces and thin films

## Abstract

Here, we show that metal oxide surfaces catalyze the formation of intermediate defluorinated tetrafluoroethylene (TFE) radicals, resulting in enhanced binding on the corresponding metal oxide surfaces. We attribute the preferential adsorption and radical formation of TFE on Cr_2_O_3_(0001) relative to TiO_2_(110) to the low oxygen coordination of Cr surface atoms. This hints at a possible dependence of the TFE binding strength to the surface stoichiometry of metal-oxide surfaces.

## Introduction

Being able to join dissimilar materials (cf., e.g., Refs.^1–4^ and references therein) is a key enabling technology to innovative and sustainable materials design for industrial applications. Some notable examples include: polymer-metal composites for bio-prosthetics and medical tools^1,2^; polymer-functionalized metal oxide surfaces for specialized applications^3–7^; polymers passivating metal oxide defects to increase carrier efficiency for better optoelectronic materials^8,9^; and polytetrafluoroethylene (PTFE) used as fluorine sources to form oxyfluoride surfaces and functionalize metal-oxides towards the realization of superconductors^10^. All of these applications fundamentally start with polymer adhesion on metal surfaces.

Two of the most commonly used metals for industrial applications are titanium and stainless steel, due to their notable physical properties, e.g., being lightweight and less susceptible to corrosion. In actual applications, these metals are exposed to oxidizing agents in the environment such as O_2_ or water vapor, hence, they still manifest a thin layer of metal oxide surface. For example, on stainless steel surfaces, a layer of Cr_2_O_3_ forms as a protective coating against further oxidation^11^. Similarly, TiO_2_ thin layers form on the surface of titanium, enhancing its biocompatibility for medical purposes^12^. Studies also show that the formation of thin metal oxide surfaces enhances binding to other metals and insulating polymers through welding or irradiation of the surface^11–15^. Here, we show the role of the reactivity of these thin metal oxide films to chemically bind with TFE.

In the following, we present results of our study on the adsorption of tetrafluoroethylene (TFE) on TiO_2_(110) and Cr_2_O_3_(0001). We found TiO_2_(110) inert and Cr_2_O_3_(0001) active to TFE (molecular) adsorption. This can be attributed to the nature of the surface metal atoms and the corresponding oxygen coordination. Furthermore, we found that defluorination of TFE promotes adsorption on both TiO_2_(110) and Cr_2_O_3_(0001). These results indicate the role of the surface as a catalyst to form intermediate TFE radicals and promote adsorption on metal-oxide surfaces. Thus, the possibility of joining dissimilar materials (in this case polymer and metal-oxide surface).

## Results and discussions

### Molecular Adsorption of TFE on TiO_2_(110) and Cr_2_O_3_(0001)

**Figure 1:**
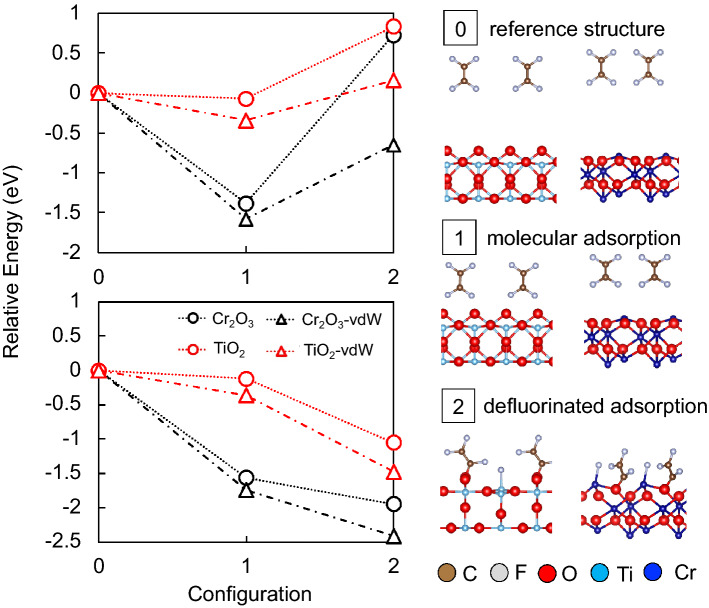
TFE on TiO_2_(110) and Cr_2_O_3_(0001) in 3 different configurations, viz., reference structure (0), molecular adsorption (1), and defluorinated adsorption (2) on the corresponding surfaces. Upper panel corresponds to the relative energies of optimized adsorbates on frozen surfaces. Lower panel corresponds to the relative energies with surface relaxation. (Note stronger TFE adsorption on Cr_2_O_3_(0001) than on TiO_2_(110), having retained energy trend after implementing van der Waals (vdW) correction).

In Fig. 1, we see weak (ca. − 0.07 eV, Configuration 1) molecular adsorption of TFE monomer on TiO_2_(110) and strong (ca. − 1.38 eV, Configuration 1) adsorption on Cr_2_O_3_(0001). We find the adsorbed TFE retaining its planar structure, negligibly modified by TiO_2_(110). These results and observations could be compared with previous studies showing an inert TiO_2_ towards fluorination from PTFE forming surface oxyfluorides^10^. On the other hand, we find a relatively stronger binding for TFE adsorbed on Cr-terminated Cr_2_O_3_(0001), with the molecular plane tilted relative to the surface axis. We found that molecular adsorption of TFE on both metal oxide surfaces does not result in any significant relaxation of the surface. However, the difference in the adsorption energy could be attributed to the difference in the surface oxygen (O)-coordination of the surface metal atoms (Ti and Cr).

**Figure 2:**
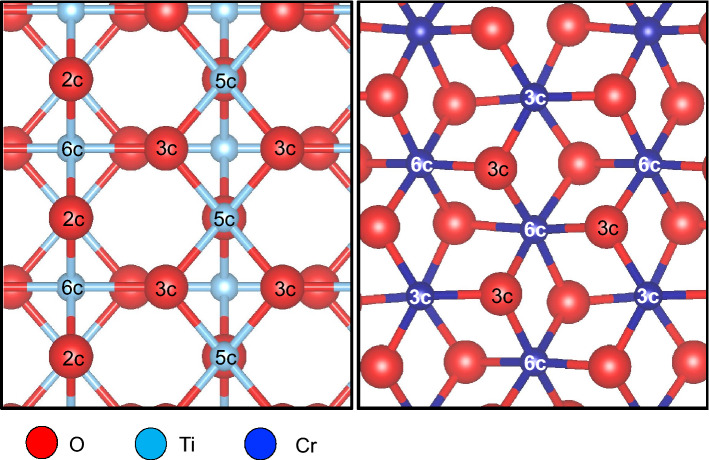
Top view of TiO_2_(110) (left panel) and Cr_2_O_3_(0001) (right panel), with coordination numbers of surface and subsurface atoms indicated. Note the lower coordination number of the surface Cr atoms as compared to the surface Ti atoms.

In Fig. 2, by inspection, we see that the surface Ti on TiO_2_(110) have higher O-coordination than the surface Cr on Cr_2_O_3_(0001). We attribute the difference in surface reactivity, i.e., adsorption preference, to the difference in surface metal–oxygen ratio. We define this ratio as the number of low coordinated surface metal ions to the fractional number of oxygen atoms bound to it, i.e., 3:7 for TiO_2_(110) and 1:1 for Cr_2_O_3_(0001). To verify this, we have added an additional Cr termination on the surface of Cr_2_O_3_(0001) (4:3 Cr to O ratio) and found a stronger adsorption of TFE with a pronounced non-planar geometry. As expected, we can enhance TFE adsorption on TiO_2_(110) by introducing oxygen vacancies (cf., e.g., Refs.^16–18^, and references therein).

**Figure 3:**
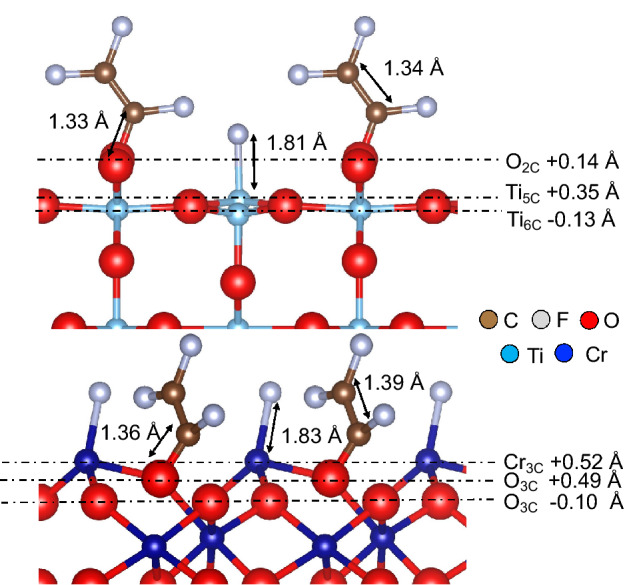
Optimized structure for defluorinated TFE adsorption with the corresponding surface relaxation after adsorption. (+) refers to relaxation of surface atoms towards the vacuum and (−) refers to relaxation of surface atoms towards the bulk. The values are deviations from the clean surface configuration.

It requires energy to break the C–F bond of TFE and, in Fig. 1, we see an endothermic dissociative adsorption of TFE (i.e., Configuration 2, with dissociated C–F bond) on both TiO_2_(110) and Cr_2_O_3_(0001), with respect to the molecular state (Configuration 1). However, upon surface relaxation, the total energy lowers, resulting in a rather exothermic adsorption for C_2_F_3_ + F on both oxide surfaces (cf., Fig. 1). As mentioned earlier, such surface relaxations are negligible in TFE molecular adsorption. The binding of C_2_F_3_ on surface O atom and the binding of F on surface metal atom (Ti and Cr) resulted in an upward (coordinate) shift of the interacting surface atoms. By comparison, we can see a greater upward shift of Cr and O towards the vacuum for Cr_2_O_3_, whereas a relatively smaller relaxation on TiO_2_ upon adsorption of the defluorinated TFE (cf., Fig. 3). (Note that the energies from Configuration 0 to 2 on both TiO_2_(110) and Cr_2_O_3_(0001) lowers after considering van der Waals correction (vdW-DFT-D2) in the calculation, as it is expected. Still, the energy trend remains (stronger binding on Cr_2_O_3_ than on TiO_2_).

**Figure 4:**
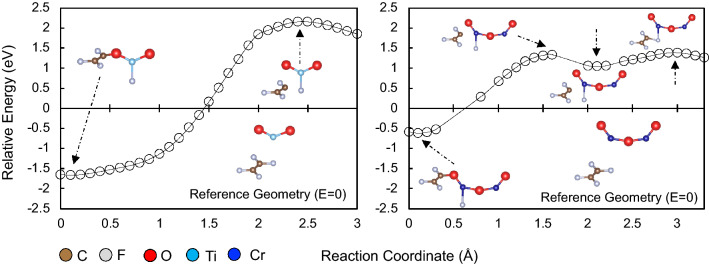
Energies [eV] required to dissociate one F from TFE in the presence of TiO_2_ (left panel) and Cr_2_O_3_ (right panel). Energies given for different configurations (reaction coordinate) relative to the corresponding reference geometries in the insets (*E*=0). Note that it requires 5.3 eV to dissociate one F from TFE in vacuum.

The relative energy plots suggest that the presence of the metal oxide surfaces lowered the energy needed to break the TFE C–F bond. Note that it requires 5.3 eV to dissociate one F from TFE in vacuum. To explore the possibility of a lowered TFE C–F bond dissociation barrier in the presence of metal-oxides, we implemented a simple dissociation model of TFE using the molecular counterpart of the metal-oxide surfaces. In Fig. 4, we show the calculated potential barriers from the molecular TFE state to the dissociated TFE state on Cr_2_O_3_ (ca. 1.39 eV) and TiO_2_ (ca. 2.16 eV). It can be seen from the simple molecular model that C–F dissociation energy lowers in the presence of metal-oxides. These results indicate the role of the surface as a catalyst to form intermediate defluorinated TFE radicals. In the following, we focus on the adsorption of defluorinated radicals of TFE on Cr_2_O_3_ and TiO_2_ surfaces.

### Adsorption of defluorinated radicals of TFE on TiO_2_(110) and Cr_2_O_3_(0001)

**Figure 5:**
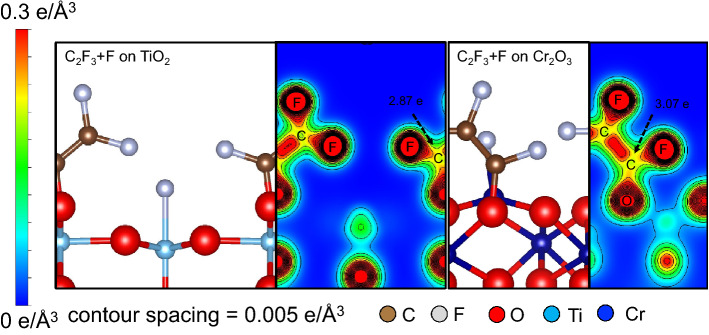
Charge density distributions for TFE adsorbed (dissociated) as C_2_F_3_ and F on TiO_2_(110) (left panel) and on Cr_2_O_3_(0001) (right panel). A higher accumulation of charge about the C_2_F_3_ C=C observed on Cr_2_O_3_(0001).

**Figure 6:**
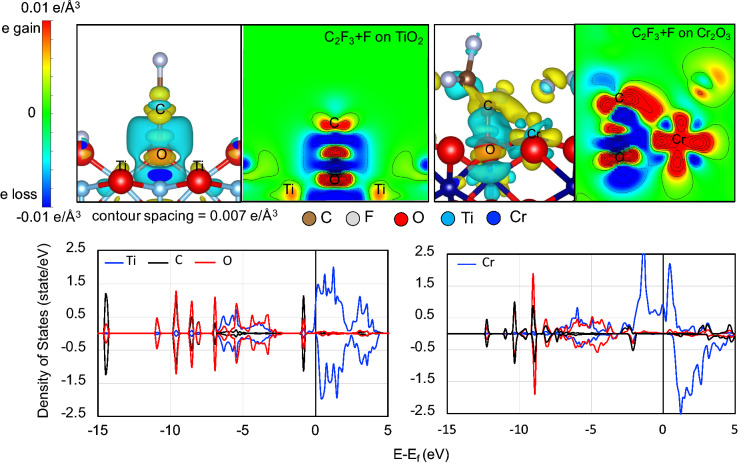
Charge density difference for C_2_F_3_ + F on TiO_2_(110) (upper left panel) and Cr_2_O_3_(0001) (upper right panel). Yellow to red region indicates electron gain. Light blue to dark blue region indicates electron loss. Projected density of states for C_2_F_3_ + F on TiO_2_(110) (lower left panel) and Cr_2_O_3_(0001) (lower right panel).

Upon defluorination (cf., e.g., Fig. 1, Configuration 2), the C_2_F_3_ creates a new bond with surface O atoms and the dissociated F atom adsorbs atop the adjacent transition metal atom. We also see a relatively more stable adsorption on Cr_2_O_3_(0001) than on TiO_2_(110). This results in a higher charge population around the carbon end of C_2_F_3_ on Cr_2_O_3_(0001) than on TiO_2_(110) (cf., Fig. 5). The relatively higher accumulation of charge from Cr_2_O_3_ (0.2 e higher) results in a longer C=C bond length (shown in Fig. 3) as compared to that on TiO_2_. From the corresponding charge density difference distribution (cf., Fig. 6) electron contribution comes from both surface (oxygen and metal) atoms. We see a more pronounced participation of Cr in TFE radical bonding as compared to Ti shown by the charge gain region (yellow region) between C and Cr surface atom. By plotting the projected density of states (PDOS), after TFE radical bonding, we show a strong hybridization of the C *p* states with the *d* electrons of Cr. This is less evident in the case of TiO_2_ where hybridization is mainly through the surface oxygen atom. As mentioned in the previous section, the surface metal–oxygen ratio influences metal-oxide surface reactivity towards TFE adsorption. From the TiO_2_(110) geometry, we find the first Ti layer completely enclosed by the octahedral cage of O, resulting in a low surface Ti–O ratio. This accounts for the weak interaction of surface Ti towards C_2_F_3_. Next, we show in Table 1 the corresponding adsorption energies of CF, CF_2_, CF_3_, CF_4_, C_2_F, C_2_F_2_, C_2_F_3_ on TiO_2_(110) and Cr_2_O_3_(0001). In general, defluorinated TFE radicals with intact C=C bond show stronger adsorption, and preference for adsorption on Cr_2_O_3_(0001). We also show that in most cases, radicals with low fluorine content manifest stronger binding on the oxide surfaces. These results indicate that chemical adsorption of the TFE monomer starts with defluorination and adsorption with an intact C=C.

**Table 1:** Adsorption energy of defluorinated TFE radicals on Cr_2_O_3_(0001) and TiO_2_(110).

TFE Radical	*E*_ad_ [eV] on TiO_2_(110)	*E*_ad_ [eV] on Cr_2_O_3_(0001)
CF	− 2.05	− 4.48
CF_2_	− 0.47	− 2.75
CF_3_	− 1.66	− 2.2
CF_4_	− 0.05	− 0.63
C_2_F	− 2.34	− 5.31
C_2_F_2_	− 2.42	− 5.07
C_2_F_3_	− 2.02	− 2.53

## Summary and conclusion

In summary, we have shown that defluorination is necessary to increase chemical bonding between tetrafluoroethylene (TFE) on TiO_2_(110) and Cr_2_O_3_(0001). The metal oxide surface catalyzes defluorination, resulting in the formation of intermediate radicals that bind strongly to the corresponding metal oxide surfaces. As expected, the reactivity of the corresponding metal oxide surfaces depends on the oxygen coordination of metal surface atoms. The surface Cr on Cr_2_O_3_(0001) has a lower fractional oxygen coordination as compared to the surface Ti on TiO_2_(110). As a result, we find stronger bonding of TFE on Cr_2_O_3_(0001) than on TiO_2_(110). This also indicates that introducing oxygen vacancies (cf., e.g., Ref.^16–18^, and reference therein), and non-ionizing radiations (cf., e.g., Ref.^19^ and references therein) to form intermediate radicals could promote binding of polymers to metals. These results should provide insights for better materials design, specifically towards polymer adhesion on metal-oxide surfaces.

### Computational method

To study the adsorption of TFE and its fragments on TiO_2_(110) and Cr_2_O_3_(0001), we performed density functional theory^20,21^ (DFT)-based total energy calculations^22–26^ using projector augmented wave (PAW) formalism and plane wave basis set (cutoff energy of 550 eV), and Perdew–Burke–Enzerhof (PBE) generalized gradient (GGA) exchange correlation functionals^27,28^. We adopt the Monkhorst and Pack method to perform the Brillouin zone integrations, with (9 × 9 × 1) special *k*-points^29^. To model TiO_2_(110) and Cr_2_O_3_(0001), we used periodically repeated slabs of (2 × 1) and (1 × 1) surface unit cells, respectively, separated by 15 Å thick vacuum region along the surface normal. The lattice constant obtained upon structural optimization for Cr_2_O_3_(0001) is 5.03 Å and the lattice constants for TiO_2_(110) are 2.97 Å and 6.59 Å. These structural geometries are in good agreement with experimental and theoretical studies^30–32^. Each slab consists of 2 layers (7 atomic planes) of O-Ti–O and Cr-O_3_-Cr. In the case of Cr_2_O_3_, we used a Cr terminated surface as it was found to be more stable than other terminations^30^. We performed geometric optimization considering energy convergence of less than 10^−5^ eV and residual forces below 0.01 eV/Å. For the molecular and dissociated adsorption of TFE we implemented both frozen and relaxed surface calculations. We implemented van der Waals correction using DFT-D2 incorporated in the VASP code.
